# The Venus flytrap attracts insects by the release of volatile organic compounds

**DOI:** 10.1093/jxb/ert455

**Published:** 2014-01-13

**Authors:** Jürgen Kreuzwieser, Ursel Scheerer, Jörg Kruse, Tim Burzlaff, Anne Honsel, Saleh Alfarraj, Plamen Georgiev, Jörg-Peter Schnitzler, Andrea Ghirardo, Ines Kreuzer, Rainer Hedrich, Heinz Rennenberg

**Affiliations:** ^1^Professur für Baumphysiologie, Institut für Forstwissenschaften, Albert-Ludwigs-Universität Freiburg, Georges-Köhler-Allee Geb. 053/054, 79110 Freiburg, Germany; ^2^Professur für Forstzoologie und Entomologie, Institut für Forstwissenschaften, Albert-Ludwigs-Universität Freiburg, Tennenbacher Strasse 4, 79085 Freiburg, Germany; ^3^Zoology Department, College of Science, King Saud University, Riyadh 11451, Saudi Arabia; ^4^Fly Facility, Max Planck Institute of Immunobiology and Epigenetics, Stübeweg 51, 79108 Freiburg, Germany; ^5^Research Unit Environmental Simulation (EUS), Institute of Biochemical Plant Pathology, Helmholtz Zentrum München, Ingolstädter Landstr. 1, 85764 Neuherberg, Germany; ^6^Lehrstuhl für Botanik I, Julius-von-Sachs-Institut für Biowissenschaften, Julius-von-Sachs-Platz 2, 97082 Würzburg, Germany

**Keywords:** Carnivorous plants, *Dionaea muscipula*, *Drosophila melanogaster*, nitrogen status, olfactory bioassay, plant–animal interaction, VOC emissions.

## Abstract

Does *Dionaea muscipula*, the Venus flytrap, use a particular mechanism to attract animal prey? This question was raised by Charles Darwin 140 years ago, but it remains unanswered. This study tested the hypothesis that *Dionaea* releases volatile organic compounds (VOCs) to allure prey insects. For this purpose, olfactory choice bioassays were performed to elucidate if *Dionaea* attracts *Drosophila melanogaster.* The VOCs emitted by the plant were further analysed by GC-MS and proton transfer reaction-mass spectrometry (PTR-MS). The bioassays documented that *Drosophila* was strongly attracted by the carnivorous plant. Over 60 VOCs, including terpenes, benzenoids, and aliphatics, were emitted by *Dionaea*, predominantly in the light. This work further tested whether attraction of animal prey is affected by the nutritional status of the plant. For this purpose, *Dionaea* plants were fed with insect biomass to improve plant N status. However, although such feeding altered the VOC emission pattern by reducing terpene release, the attraction of *Drosophila* was not affected. From these results it is concluded that *Dionaea* attracts insects on the basis of food smell mimicry because the scent released has strong similarity to the bouquet of fruits and plant flowers. Such a volatile blend is emitted to attract insects searching for food to visit the deadly capture organ of the Venus flytrap.

## Introduction

Among the estimated quarter of a million vascular plants on earth (Heywood, 1995), only somewhat over 600 species exhibit a carnivorous lifestyle (Ellison and Gotelli, 2009). Botanical carnivory has evolved at least six times among the angiosperms, and representative species are found in 11 families of five orders: Poales, Caryophyllales, Oxalidales, Ericales, and Lamiales (Ellison and Gotelli, 2009). Carnivorous plants are usually abundant in bright and wet habitats, which are characterized by very poor nutrient availability (Givnish *et al.*, 1984). Only under such extreme marginal environmental conditions does the benefit of nutrient gain by cost-intensive carnivory seem to provide an advantage compared to a noncarnivorous lifestyle (see cost–benefit model by Givnish *et al.*, 1984; see also Ellison and Gotelli, 2001, 2009; Ellison, 2006; Kruse *et al.*, 2013).

The carnivorous plant *Dionaea muscipula*, together with *Drosera* species, belongs to the family Droseraceae*. Dionaea* is endemic in the nutrient-poor, flooding-prone pocosin wetlands in North and South Carolina in the USA. By catching insects, *Dionaea* and other carnivorous plants improve their nutrient status, particularly that of nitrogen, thus stimulating growth (Ellison, 2006). *Dionaea muscipula* displays a particular fascinating adaptation to carnivory, already recognized by Darwin (1875). Unlike other carnivorous plants that passively trap prey animals by sticky surfaces (e.g. *Drosera*) or with the pitcher trap (*Nepenthes*), the Venus flytrap actively catches small animals with specialized capture organs. Prey animals touching the mechanosensory organs located in the inner parts of the snap traps induce an action potential which—in interplay with plant hormones—eventually causes the closure of the traps (Volkov *et al*., 2007, 2008; Escalante-Pérez *et al.*, 2011). Such snapping presents one of the fastest movement in plants (Volkov *et al.*, 2008); the ‘rapidity and force of movement’ of these traps led Darwin (1875) to designate the Venus flytrap as ‘one of the most wonderful plants in the world’. The Venus flytrap has a very broad size spectrum of their prey (Gibson, 1991; Gibson and Waller, 2009; Hutchens and Luken, 2009). Small animals (around 2mm long) get mainly caught by small young traps while larger prey are caught in the more mature capture organs. *Dionaea muscipula* ‘hunts’ nonflying animals from the families Araneae and Formicidae as well as flying animals of the orders Coleoptera and Diptera (Jones, 1923; see also Ellison and Gotelli, 2009).

Almost 140 years ago, Charles Darwin already raised the question whether there is a particular prey attraction mechanism in *Dionaea muscipula*. A possible mode of insect attraction could be shape and colour, as was demonstrated in earlier studies (Joel *et al.*, 1985; Ichiisi *et al.*, 1999; Schaefer and Ruxton, 2008). Jürgens *et al*. (2009) hypothesized that luring prey via emission of volatile organic compounds (VOCs) might be a further principle and found trace emissions of some VOCs from isolated traps of the Venus flytrap. Clear evidence for attraction of *Drosophila* flies and an ant species via release of VOCs was demonstrated for the passive carnivorous plant *Nepenthes rafflesiana* (Di Giusto *et al.*, 2010). Plants emit a multitude of more than 1700 VOCs from organs such as fruits and vegetative and floral tissues (Pichersky and Gershenzon, 2002; Knudsen and Gershenzon, 2006; Rowan *et al.*, 2009; Gonzalez-Mas *et al.*, 2011). VOCs often act as signalling molecules for plant–animal or plant–plant communication (Dudareva *et al.*, 2013). The release of volatile scents is often connected to plant reproduction, particularly attraction of pollinators to flowers (Raguso, 2008) and seed dissemination (Hodgkison *et al.*, 2007; Borges *et al.*, 2008; Youngsteadt *et al.*, 2008). Flowers, for example, typically emit a mixture of 20–60 different volatile chemical structures (Knudsen and Gershenzon, 2006). The resulting floral scent signals the maturity state of the flower to the pollinator. Individual blends seem even allow animals to discriminate between different plant species (Dudareva *et al.*, 2013). In addition, VOCs are involved in the protection against abiotic stress (Loreto and Schnitzler, 2010) and in defence reactions against herbivores (Arimura *et al.*, 2005; Degenhardt *et al.*, 2009; Unsicker *et al.*, 2009; Ali *et al.*, 2012).

The present study tested the hypothesis that *Dionaea muscipula* emits an insect-attracting volatile scent to guide prey towards the capture organs of the active carnivore. Besides the analysis of the volatile compounds emitted by the Venus flytrap, this work performed olfactory bioassays to elucidate if insects (here *Drosophila melanogaster*) are indeed attracted by the volatile scent. It was further hypothesized that the Venus flytrap produces the energy-costly volatile compounds only under conditions of nitrogen deprivation. To test this assumption, *Dionaea muscipula* plants were fed with insect biomass and the VOC emissions of such fed plants were compared with nonfed plants.

## Materials and methods

### Plant and animal material

*Dionaea muscipula* plants were obtained by vegetative propagation of root rhizomes and were purchased from a commercial gardener. The plantlets were cultivated for 3–4 months under greenhouse conditions at 24±4 °C. During that time they were supplied with a slow-release fertilizer and rainwater. For adaptation to the experiments, the plants were transferred into plastic pots (350cm^3^) containing nutrient-poor peat as a substrate. Therefore, *Dionaea muscipula* plants had very limited access to mineral nutrients during the course of the experiments, resulting in low N status (total N content <1% of dry weight; Kruse *et al.*, 2013). The plants were placed into a climate-controlled growth chamber (HPS 1500, Heraeus Vötsch, Hanau, Germany) and exposed to long-day conditions under a 16/8 light/dark cycle (photosynthetic photon flux density, PPFD, 350 μmol m^–2^ s^–1^, 25/20 °C, relative humidity 70%. The photosynthetically active leaves of the plants were pale green; the inner zones of the traps were bright red. Plants had an average trap-to-petiole biomass ratio of 3.2 (Kruse *et al.*, 2013). For experiments, plants of similar size were used with an average leaf area of 35.9±4.7cm^2^.

Fruit flies (*Drosophila melanogaster*) of the Oregon white-eye type (Bloomington *Drosophila* Stock Center) were used as model prey system in the present experiments. Flies were reared on standard medium (l^–1^: 79g agar, 275g Torumel yeast, 520g corn meal, 1000g granulated sugar, 23.8g Nipagin M) in a laboratory incubator under controlled conditions (12/12h light/dark cycle, 25±1 °C, relative humidity 50–60%. A short pulse of CO_2_ was given to immobilize 12–24-h-old flies before sorting and transferring them into fresh vials containing the food source (10 males or 10 females per vial). All animals used in the experiments were 24–96-h-old; all females were virgins. As in other olfactory studies with *Drosophila melanogaster* (e.g. Becher *et al.*, 2010), prior to use in experiments, males and females were starved for 24 and 48h, respectively, by placing them into vials without any food source; water, however, was provided during this starving period.

### Feeding experiment

For feeding *Dionaea muscipula* plants with insects, this work produced a stock of insect powder. This procedure was chosen in order to standardize the feeding approach and supplying the plants with exactly the same amount of insect biomass. The powder was produced from caterpillars of the moth *Creatonotos transiens*, a member of the Arctiidae family. For preparing the powder, *C. transiens* larvae were fed with an artificial diet (Bergomaz and Boppré, 1986). In addition, larvae were given access for 3 d to glass fibre discs impregnated with monocrotaline as feeding stimulant (Boppré and Schneider, 1989). Thereafter, drying, homogenizing, and pooling the larvae produced a homogeneous powder containing 10.3±0.1% nitrogen. For feeding *Dionaea muscipula* plants, 20mg powder suspended in 100 μl water was applied to the plants’ traps (Kruse *et al.*, 2013).

### Olfactory bioassays

To test whether *Dionaea muscipula* released a scent that was able to attract insects, Y-tube olfactory choice bioassays were performed. For this purpose, a system was constructed consisting of a source of VOC-free synthetic air (containing 400 ppm CO_2_ and 21% O_2_ in N_2_; Air Liquide, Germany), two glass cuvettes (3 l), and a Y-tube where the fruit flies were inserted. The air was humidified to approximately 40% relative humidity by flushing it through a glass bottle filled with demineralized H_2_O. The air stream was then split in two halves and led into the cuvettes at defined flow rates of 1 l min^–1^.

For bioassays, the cuvettes were kept empty as a control or contained either a sample of fruit fly food or one *Dionaea muscipula* plant. The plants were either kept potted in the peat substrate or were carefully taken out of the pots without damaging the root system, rinsed with water to remove peat particles, and then placed into a beaker containing demineralized water. In further trials, both cuvettes contained either a fed or an unfed plant potted in the peat substrate. The cuvette outlets were connected with Teflon tubing to the two upper arms of the Y-tube, which was also made of glass. The basal arm and each of the upper arms had a length of 50cm and an internal diameter of 2.5cm. Air speed in each upper arm of the Y-tube was 3.4cm s^–1^ and in the basal arm was 6.8cm s^–1^. Importantly, cuvettes (in a climate-controlled chamber) and Y-tube (in a curtain-enclosed room in front of the climate chamber) were spatially and visually separated, preventing the fruit flies from seeing the cuvettes or using any visual cues for orientation. During bioassay experiments, temperatures in the cuvettes were adjusted to 30 °C and plants were either exposed to light (500 μmol PPFD m^–2^ s^–1^) or to darkness. The separate room containing insects and Y-tube was adjusted to 25 °C and light intensities of 400–500 μmol m^–2^ s^–1^. The light was homogeneously distributed to prevent any optical orientation for the insects towards the light. These conditions were chosen to ensure standardized and close to natural conditions for the plants (30 °C) and no stress conditions for the *Drosophila* flies (25 °C).

To start the experiments, individual starved male or female fruit flies were inserted into the system at the bottom of the basal arm and movement was observed for 3min. After every 8–10 insect trials, the left and right arms of the Y-tube were changed and the plant in the cuvette was replaced by a new one. To avoid any memory effects by interaction of ‘sticky’ polar oxygenated compounds with cuvette surfaces, the cuvettes were cleaned after each set of trials and plants were alternatively put into the right or the left cuvette. Consequently, between four and eight different plants were used as an odour source per treatment. According to their behaviour, the flies were divided into three groups: (i) movement to the left arm, (ii) movement to the right arm, (iii) no movement/decision. To be classified into (i) or (ii), flies had to stay at least for 30 s in the respective arm.

### VOC emission studies

#### Experimental set up

To study VOC emissions from *Dionaea*, the dynamic cuvette system described by Kreuzwieser *et al.* (2001) was applied. This system consisted of two cylindrically shaped glass cuvettes with a spherical cap (total volume 1 l each, diameter 10cm, height 15cm). Both cuvettes were placed in a phytochamber of the Helmholtz Zentrum München (Neuherberg, Germany) and run in parallel using the measuring system described elsewhere (Ghirardo *et al.*, 2010, 2011). Light intensities inside the cuvette were set to a PPFD of 500 μmol m^−2^ s^−1^ (measured by light sensor LI-250A, Licor, Lincoln, NE, USA) during the light phase. Leaf temperatures of 34±1 °C during day and 21±1 °C during night were measured continuously with a thermocouple touching the bottom of the middle leaf. The cuvettes were flushed with humidified (15,000 ppm H_2_O), synthetic VOC- and O_3_-free air (400 ppm CO_2_, 21.0% v/v O_2_ in N_2_, BASI Schöberl, Germany) at defined flow rates of 500ml min^–1^ in order to decrease VOC background and avoid degradation of plant-emitted VOC. Cuvette air was homogeneously mixed with electrical fans installed in the enclosures. For plant VOC emission measurements, each cuvette was hosting either a fed or an unfed *Dionaea muscipula* plant potted on soil substrate; four plants were used per treatment. To correct emission rates for a possible VOC background, several control measurements were performed, including (i) pots with soil substrate only (without plant) and (ii) five 1.5-ml glass tubes, each filled with 20mg insect powder suspended in 100 μl distilled water (representing the amount added into the traps of fed plants). The low emissions from these controls were subtracted from the emissions of the potted *Dionaea muscipula* plants.

#### Online mass spectrometry

Air leaving the cuvettes was drawn at a flow rate of 150ml min^−1^ throughout short (<1.5 m), heated, and thermally isolated stainless steel lines to a proton-transfer-reaction mass-spectrometer (PTR-MS, Ionicon Analytik, Austria) for online analysis of VOCs. The details of the PTR-MS operating parameters and analytical procedures used in the present study are given elsewhere (Ghirardo *et al.*, 2010). Briefly, the PTR-MS was run with the following parameters: E/N = 110 Td (E = electric field; N = number of density of the drift tube molecules; Td = 1 Townsend = 10^−17^ cm^2^ V molecule^−1^), p-drift = 1.73 mbar, T = 43 °C, V = 400, O_2_^+^ and NO^+^ < 2% of H_3_O^+^. Calibration of methanol and monoterpene were directly achieved using standards inside a mixture of 11 VOC (Apel-Riemer Environmental, Denver, CO, USA). Quantification of products of the lipoxygenase (LOX) reaction (m99 and m101) and sesquiterpenes (m205) were achieved using the transmission factor (Hansel *et al.*, 1995) of monitored integer ion masses and the fractions of these integer ions to the fragmentation pattern of the compound obtained by pure standard. The m/z mass of 135 for the irregular monoterpene *p*-cymene was not included. Because this work used synthetic, ozone-free air, particular degradation of the labile sesquiterpenes such as caryophyllene was not expected; this assumption was supported by the results based on measurements of the calibration gas in the cuvette system. To correct raw data for the effect of water vapour pressure on fragmentation patterns (Tani *et al.*, 2004), a humidity-dependent calibration (1–50 ppb gas standards with 5000–15,000 ppm H_2_O) was performed with a VOC mix standard in N_2_ (Apel-Riemer Environmental, Denver, CO, USA). This was needed as the water vapour pressure in the cuvette changes between light and dark because of changing temperatures in the cuvette and light-dependent differences in transpiration of the plants. Calculation of VOC emission rates were normalized per projected leaf area. The limit of detection was calculated as twice the standard deviation of the count per second signal of background measurements of a pot with peat substrate divided by the instrument’s sensitivity (count s^–1^ ppb^–1^; Ghirardo *et al.*, 2010).

#### Offline GC-MS analysis

PTR-MS analysis allows online VOC quantification at high temporal resolution without differentiation of isomeric compounds (Ghirardo *et al.*, 2010). To identify the VOCs and separate different monoterpene and sesquiterpene isomers, GC-MS analysis was performed by trapping 4 l of air leaving the cuvettes onto polydimethylsiloxane-foam-adsorbent tubes (Gerstel, Mülheim an der Ruhr, Germany) at flow rates of 100ml min^−1^. This procedure was optimized for determination of nonpolar compounds; some polar volatiles such as methanol, ethanol, and C6 LOX products were not included in this analysis. Samples were analysed after thermal desorption and cryofocusing by GC-MS, as described by Ghirardo *et al.* (2012). VOCs from control experiments were used for background subtraction. For the quantification of VOCs, individual response factors were determined using the total ion count from calibration curves (*R* ^2^>0.98) of pure standards (α-pinene, sabinene, 3-carene, *p*-cymene, limonene, linalool, *trans*-β-caryophyllene, α-farnesene, and nerolidol) at four different concentrations (1–100 pmol (l hexane)^–1^). Other monoterpenes not present in the standard were quantified using sabinene; other monoterpene alcohols using linalool; other sesquiterpenes using (−)-β-caryophyllene; and other sesquiterpene alcohols using nerolidol. For the quantification of aliphatic and aromatic compounds, a response factor was calculated for each compound by using the response factor of sabinene (*R* ^2^>0.99) and was normalized based on molecular weight in order to consider the changes of total ion count responses due to different molecular masses. The same procedure was used for the quantification of the aliphatic and aromatic alcohols, except that the response factor of the linalool standard (*R* ^2^>0.99) was used as reference.

### Statistics

The blends of VOCs emitted from plants were analysed by a multivariate data analysis approach, using principal component analysis (PCA) and partial least square regression (PLSR) statistical methods. Both analyses were performed using the software package SIMCA-P version 13.0.0.0 (Umetrics, Umeå, Sweden). For the analysis, VOC emission rates from each individual biological replicate were used as X. Before PCA and PLSR analyses, data were preprocessed by log transformation [X = log(X+1)], mean centred, and scaled to unit variance. Cross-validation was used to validate the number of significant PCA and PLSR components (Eriksson *et al.*, 2006) using a 99% confidence level on parameters and seven cross-validation groups. PCA was performed to describe the different blends of VOC emitted from plants, in an objective and unsupervised manner. PLSR was then performed using the regression type PLS of SIMCA-P. The Y-variable was chosen to describe if the samples were fed (Y = –1) or unfed (Y = 0). PLSR was validated using analysis of variance testing of cross-validated predictive residuals (CV-ANOVA; Eriksson *et al.*, 2008). The overall analysis aimed to identify which compound and at which degree the VOC was positively or negatively correlated to plant feeding with insect powder. Therefore, the putative volatile compound determining the VOC profile of fed and unfed plants was classified significantly important when this volatile had both importance in the projection (VIP) <1 and the uncertainty bar computed by jack-knife method (Efron and Gong, 1983) was smaller than its respective VIP value. Additionally, statistically significant differences (*P*<0.05) between VOC emission rates of different types of plants (fed vs. nonfed) were determined independently from multivariate data analysis with the Tukey’s test under ANOVA. Results of the choice bioassays were analysed with a binomial test. Fruit flies making no decision were excluded from the analysis.

## Results

**Fig. 1.**
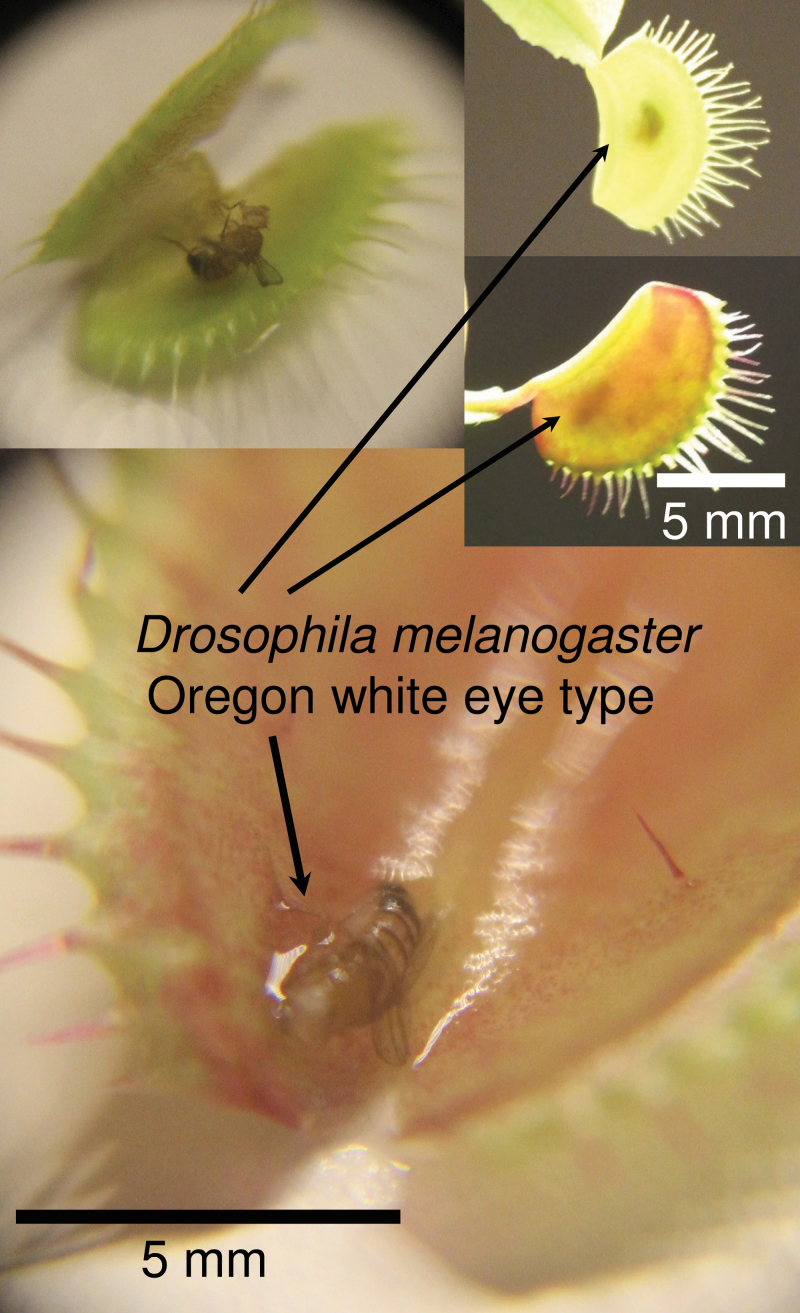
*Drosophila melanogaster* caught by *Dionaea muscipula* snap traps. Closed traps containing fruit flies and traps opened carefully with a scalpel.

The present study used *Drosophila melanogaster* as an insect model and tested whether the volatile scent emitted from *Dionaea muscipula* attracted this species. Consistent with results of Gibson (1991), *Dionaea muscipula* was able to capture *Drosophila* flies (Fig. 1). Shortly after placing hungry *Drosophila* flies and *Dionaea* plants together into one enclosure, the flies were observed visiting the snap traps. It was clearly visible that the mechanosensory organs in the larger traps were not stimulated even when the fruit flies were touching them repeatedly. However, the snap mechanism of younger, smaller traps was stimulated and these organs caught *Drosophila* flies very efficiently.

### *Dionaea* plants attract starved fruit flies

**Fig. 2.**
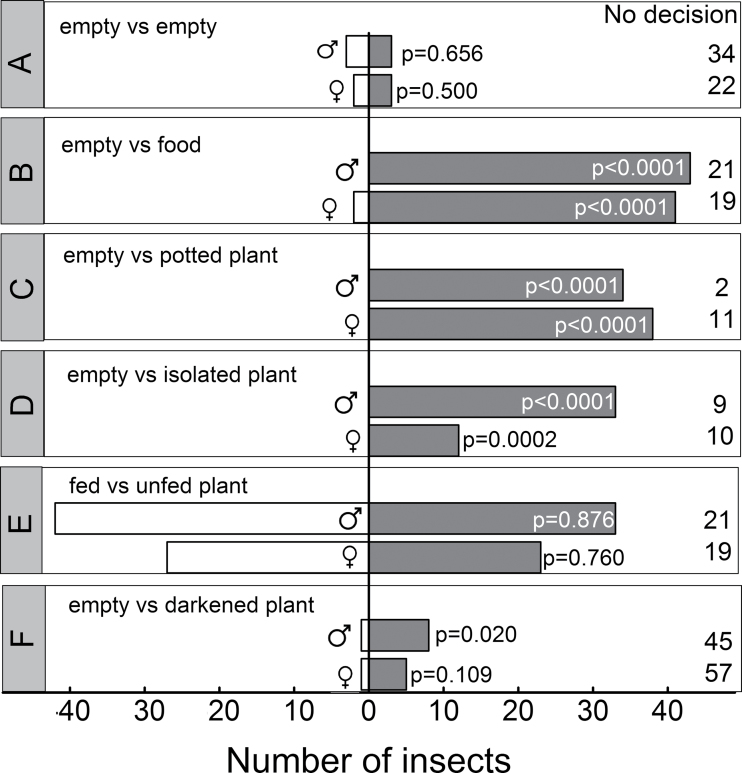
Olfactory bioassay experiments with starved male and female *Drosophila melanogaster*. (A) Both arms of the Y-tube connected to empty cuvettes; (B) one cuvette was empty and the other contained 2g *Drosophila* food; (C) one cuvette was empty and the other contained a potted *Dionaea muscipula* plant; (D) one cuvette was empty and the other contained a hydroponic *Dionaea muscipula* plant; (E) one cuvette contained an unfed *Dionaea muscipula* plant and the other cuvette a plant fed with insect powder; (F) one cuvette was empty and the other contained a *Dionaea muscipula* plant kept in the dark. Individual animals were placed in the basal arm of the Y-tube and observed for 3min. Data are total numbers of animals selecting specific cuvettes or with no decision with 8–10 insects from 4–10 independent experiments. Statistically significant differences were calculated by a binomial test.

To test whether *Drosophila melanogaster* was attracted by *Dionaea muscipula*, male and female fruit flies were placed in the basal arm of the Y-tube. Immediately after exposure into the Y-tube, insect movement towards the arm connected to the plant cuvette was observed. When the air entering the Y-tube was flushed through two empty control cuvettes, 91±12% of the starved animals showed only marginal movement and made no decision for a specific cuvette (Fig. 2A). In another series of control experiments, one of the cuvette contained the standard food source/scent of the flies. About two-thirds (67±21%) of the insects moved towards the food source when they were previously starved for 24h (males) or 48h (females); the remaining population did not decide on a cuvette; only two females moved towards the empty control cuvette (Fig. 2B). In the test for empty cuvette versus *Dionaea*-containing cuvette, 85±10% of all starved insects moved towards the plant odour, the remaining flies did not move along the tube; none of the insects decided for the empty control cuvette (Fig. 2C). *Dionaea* plants cultivated as hydroponics caused a similar insect behaviour, as 71±8% of the starved flies went for the plant odour (Fig. 2D).

In another approach, it was tested whether the plants’ N status affected the attraction of insects. For this purpose, *Dionaea muscipula* plants were fed with insect powder. This treatment resulted in a significantly improved N status of fed plants (1.1% total N of dry weight) compared to unfed plants (0.88% total N of dry weight) together with higher rates of photosynthesis and 2–3-times increased growth rates (Kruse *et al.*, 2013). However, fed and unfed *Dionaea muscipula* plants attracted *Drosophila melanogaster* in exactly the same way (Fig. 2e) irrespective of whether male or female insects were used. To test for daily patterns in plant attractiveness for insects, bioassays were performed in which *Dionaea* was kept in darkness. The high number of animals without decision (83±11%) indicated a much weaker attraction of insects by the Venus flytrap during night (Fig. 2f). Of the few insects making a decision, only male *Drosophila melanogaster* were attracted significantly by the Venus flytrap but not female insects.

### *Dionaea* releases a complex blend of volatiles

**Fig. 3.**
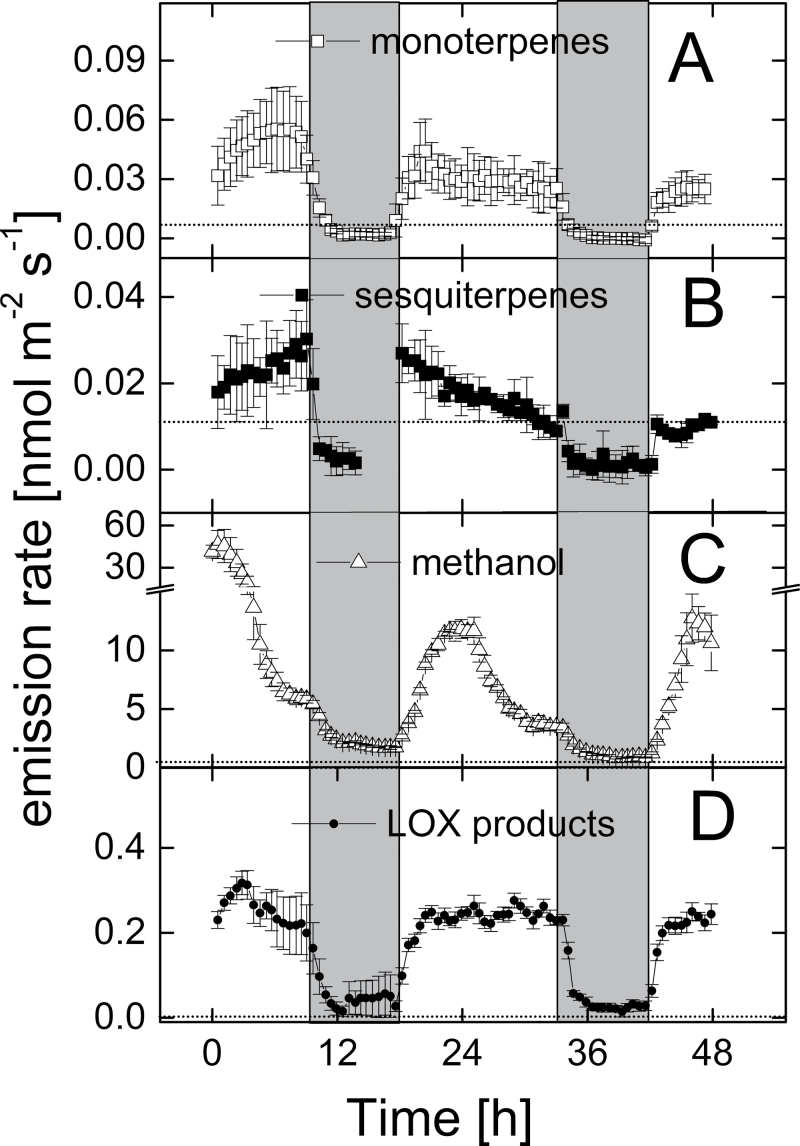
Daily pattern of the emission of monoterpenes (m137) (A), sesquiterpenes (m205; B), methanol (m33; C), and lipoxygenase (LOX) reaction products (m99+m101; D) from unfed *Dionaea muscipula* control plants. Potted plants were placed in cuvettes which were flushed with humidified synthetic air; cuvettes were set up in climate-controlled growth chambers ensuring constant environmental conditions during the experiments. VOC emission from the plants was monitored online during two subsequent days by analysing the VOC concentrations of air leaving the cuvette with a PTR-MS. Data are mean ± standard deviation of four biological replicates. The horizontal grey lines represent the individual limits of detection.

**Fig. 4.**
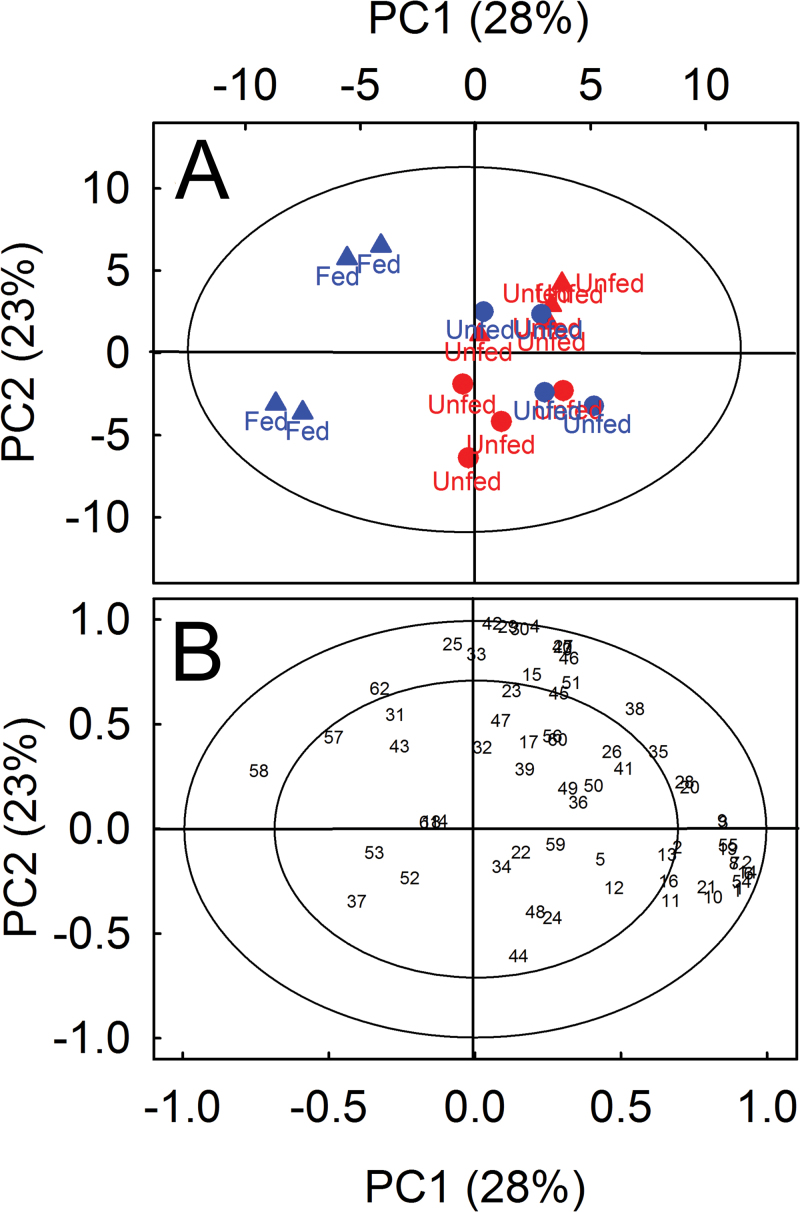
Two-dimensional score (A) and scaled-loading (B) plots of principal component analysis (PCA) computed with VOC emission rates emitted from unfed and fed *Dionaea muscipula* plants. Plants were divided into treatment group (blue) and control group (red). Filled circles indicate VOC emission rates emitted from treated and control plants at day 0 (unfed), triangles indicate their respective emission rates 4 days after, when treated plants were fed with insect powder and control plants remained unfed. The explained variance (in percentage) and the number of principal component are reported on the X- and Y-axes. Ellipse in A indicates the tolerance based on Hotelling’s T^2^ with significance level of 0.05. Numbers and letters in B reflect the compounds listed in Table 1. The outer and inner ellipses in B indicate 100 and 75% explained variance, respectively.

The bioassays clearly indicated attraction of *Drosophila melanogaster* by the Venus flytrap and suggested an important role of volatile compounds. Indeed, intact *Dionaea* plants emitted a great variety of over 60 VOCs (Table 1). These compounds comprised monoterpenes and sesquiterpenes as well as aromatic and aliphatic compounds such as alkanes, alcohols, aldehydes, and organic acids. Highest emission rates were observed for the monoterpenes *p*-cymene (>60 pmol m^–2^ s^–1^), limonene (>30 pmol m^–2^ s^–1^), and α-phellandrene (>20 pmol m^–2^ s^–1^) and for the sesquiterpene caryophyllene (>20 pmol m^–2^ s^–1^) together making up about two-thirds of total VOC emission. In addition, online monitoring of VOCs by PTR-MS showed emission of monoterpenes, sesquiterpenes, methanol, and products of the LOX reaction such as the C_6_-oxygenated compounds hexenal, hexanal, and hexenols (green leaf volatiles) with distinct diurnal patterns: high emissions during the light phase and very low emissions in darkness (Fig. 3). It was hypothesized that VOC emission from *Dionaea muscipula* is highly regulated and might be downregulated or switched off when insect attraction is no longer needed. This might be the case when *Dionaea muscipula* plants have caught prey and, as a consequence, possess a high N status. This hypothesis was tested by analysing the emission of VOCs from fed and unfed plants. Notably, N-limited, unfed plants and those fed with insect powder emitted a large number of volatiles (Table 1). The profile of VOCs released by fed plants significantly differed from the scent of unfed plants as clearly indicated by PCA (Fig. 4) and subsequent PLSR analysis (*P* = 0.040, CV-ANOVA; Supplementary Fig. S1, available at *JXB* online). PCA showed that different VOC were negatively correlated to fed plants, as indicated by the VOC found between the two ellipses (Fig. 4B, right) opposite to the fed samples (Fig. 4A, left). However, to what extent the VOC emission rates were downregulated was revealed by further PLSR analysis (Supplementary Fig. S1). The most prominent decreasing VOCs due to feeding plants were 12 monoterpenes, three sesquiterpenes, five aliphatic, and one benzenoid compounds (Table 2). Only eicosane was found to be upregulated after feeding; however, its upregulation was less important than eight other compounds which included seven terpenoids, as indicated by the lower VIP values (Table 2). The fed plants were highly negatively correlated to emission rate of sesquiterpene caryophyllene, followed by monoterpenes *trans*-verbenone, *p*-cymene, and limonene (VIP values > 1.8; Table 2). The sums of aliphatic and benzenoid compounds emitted were not found to differ significantly between fed and unfed plants (Supplementary Fig. S1 and Table 2). Taking together, plants with a high N-status (fed) quickly decreased emission rates of mainly terpenoid compounds, compared to plants with low N-status (unfed).

**Table 1.** Rates of VOC emission of unfed and fed *Dionaea muscipula* plantsVOC release was determined in ‘treatment plants’ before feeding (‘unfed-d0’) and 4 days after feeding with insect powder (‘fed-d4’); unfed control plants were also analysed at day 0 (‘unfed-d0’) and day 4 (‘unfed-d4’). Summarized are VOC with a portion ≥0.40% of the total amount of VOC emitted from plants ‘unfed-d0’. Data are mean ± SE of at least four biological replicates. Different superscript letters indicate statistically significant differences between different groups of plants (*P* < 0.05, Tukey’s test under ANOVA. Compounds highlighted by grey are responsible for the differences in the volatile blend emitted by *Dionaea muscipula* before and after insect feeding (for further statistics, see Table 2)

Compound	CAS no.	Retention time (min)	Kovats retention index	Main fragment (m/z)	Probability (%)	ID	Emission rate (pmol m^–2^ s^–1^)			
							Treatment plants		Control plants	
							Unfed-d0	Fed-d4	Unfed-d0	Unfed-d4
Sesquiterpenes										
Caryophyllene	87-44-5	36.78	1434	133	100	1	22.83±2.70^*a*^	0.39±0.25^*b*^	18.60±0.36^*a*^	20.57±2.29^*a*^
*trans*-Geranylacetone	3796-70-1	37.39	1451	43	93	2	1.58±0.34	0.64±0.06	1.17±0.45	1.25±0.20
Caryophyllene oxide	1139-30-6	41.69	1598	79	87	3	0.46±0.10^*a*^	0.00±0.00^*b*^	0.54±0.11^*a*^	0.33±0.05^*a*^
Humulene	6753-98-6	37.93	1465	161	83	5	0.20±0.16	0.05±0.05	0.29±0.10	0.20±0.06
β-Guaiene	201-860-1	38.98	1493	161	83	4	0.11±0.05	0.29±0.28	0.49±0.26	0.15±0.07
Total sesquiterpenes							25.2±3.3^*a*^	1.36±0.64^*b*^	21.1±1.3^*a*^	22.5±2.7^*a*^
Monoterpenes										
*p*-Cymene	99-87-6	19.43	1032	83	97	6	62.58±15.28^*a*^	5.27±1.31^*b*^	55.67±10.82^*a*^	63.85±14.12^*a*^
Limonene	138-86-3	19.67	1037	68	95	7	33.69±6.24^*a*^	2.16±1.51^*b*^	31.62±5.46^*a*^	31.31±7.25^*a*^
α-Phellandrene	29050-33-7	18.23	1010	121	97	8	22.69±9.13	1.18±1.12	11.45±4.39	18.74±5.41
Sabinene	3387-41-5	17.03	979	93	95	9	5.82±2.77	0.33±0.23	4.03±0.56	2.92±1.41
3-Carene	13466-78-9	18.67	1014	93	97	10	1.76±0.51	0.20±0.20	2.99±1.37	1.73±0.51
Tricyclene	508-32-7	14.91	931	93	90	11	1.36±0.83	0.07±0.07	1.65±0.64	1.59±0.63
α-Pinene	80-56-8	15.78	940	93	99	12	1.31±0.99	0.32±0.19	1.02±0.13	1.51±0.73
Camphene	79-92-5	16.06	958	93	95	16	1.14±0.76	0.06±0.06	0.91±0.11	0.90±0.34
5-Hepten-2-one,6-methyl-	110-93-0	17.35	985	108	94	17	1.01±0.18	0.90±0.21	1.18±0.33	0.91±0.17
*trans*-Verbenone	1196-01-6	29.58	1256	150	94	14	0.95±0.06^*a*^	0.00±0.00^*b*^	0.93±0.04^*a*^	0.88±0.08^*a*^
1-(1-Cyclohexen-1-yl)- ethanone	932-66-1	13.11	886	81	93	15	0.94±0.27	1.08±0.60	1.30±0.09	1.02±0.32
*cis*-para-2-Menthen-1-ol	29803-82-5	24.87	1150	93	76	19	0.52±0.08^*a*^	0.00±0.00^*b*^	0.35±0.05^*a*^	0.30±0.09^*a*^
α-Terpinene	99-86-5	19.03	1022	121	95	13	0.51±0.17	0.13±0.08	0.65±0.34	0.73±0.39
γ-Terpinene	99-85-4	20.92	1064	93	93	21	0.45±0.11	0.06±0.04	0.41±0.12	0.46±0.13
*trans*-Pinocarveol	547-61-5	24.55	1143	91	72	20	0.43±0.09^*a*^	0.08±0.06^*b*^	0.28±0.05^*a,b*^	0.15±0.07^*a,b*^
(E)-β-Ocimene	3338-55-4	20.54	1056	69	83	23	0.39±0.08	0.28±0.19	0.44±0.26	0.06±0.04
Isopinocampheol	24041-60-9	29.97	1265	70	75	24	0.11±0.06	0.03±0.03	0.16±0.09	0.06±0.06
Isomenthol	23283-97-8	30.64	1280	57	73	22	0.10±0.08	0.03±0.02	0.19±0.19	0.22±0.16
β-Linalool	78-70-6	22.79	1102	93	94	18	0.00±0.00	0.37±0.31	0.60±0.38	0.28±0.28
Total monoterpenes							136±38^*a*^	12.6±6.2^*b*^	116±25^*a*^	128±32^*a*^
Benzenoids										
Acetophenone	98-86-2	21.28	1071	105	97	25	2.51±0.38	4.13±1.41	3.58±0.14	1.87±0.21
Benzaldehyde	100-52-7	16.52	968	106	97	27	1.21±0.18	1.11±0.46	1.53±0.21	0.59±0.10
Benzyl alcohol	100-51-6	21.48	1076	108	91	26	0.56±0.34	0.49±0.35	1.73±0.61	0.48±0.27
Phenylethyl alcohol	60-12-8	9.38	769	91	74	28	0.39±0.08	0.17±0.12	0.67±0.18	0.59±0.22
Total benzenoids							4.67±0.99	5.90±2.34	7.51±1.15	3.53±0.80
Aliphatics										
Decanal	112-31-2	27.57	1208	57	97	29	11.02±3.12	16.01±6.70	19.19±3.19	6.84±0.93
Nonanal	124-19-6	23.12	1110	57	98	30	9.89±2.43	15.19±6.31	16.87±2.04	7.13±1.12
Acetone	67-64-1	5.45		43	96	38	4.19±2.06	2.05±1.51	5.69±2.20	1.91±0.71
1-Tetradecanol	112-72-1	43.21		83	96	31	3.20±0.63	3.71±0.67	1.95±0.78	1.48±0.11
n-Hexadecanoic acid	57-10-3	46.85		73	88	32	2.66±1.70	0.81±0.34	4.79±1.99	0.14±0.06
Octanal	124-13-0	18.30	1005	84	98	33	2.59±1.50	4.80±1.96	7.68±1.71	1.30±1.30
Pentadecane	629-62-9	39.25	1500	57	96	36	1.23±0.21	0.94±0.13	0.99±0.15	0.89±0.19
Acetic acid methyl ester	79-20-9	8.26	721	43	90	49	1.21±0.33	0.78±0.30	1.07±0.45	0.92±0.28
Hexanal	66-25-1	10.16	800	43	97	40	1.18±0.30	1.52±0.77	2.42±0.36	1.05±0.15
1-Octanol	111-87-5	21.30	1072	56	84	41	1.18±0.22	0.46±0.23	1.54±0.38	0.67±0.18
Heptadecane	629-78-7	43.58		57	94	34	1.10±0.12	1.06±0.22	1.38±0.53	0.99±0.08
Acetic acid	64-19-7	8.89	749	45	100	59	1.07±0.74	0.19±0.12	0.60±0.45	0.06±0.06
Undecanal	112-44-7	31.90	1310	57	97	42	1.04±0.32	1.45±0.61	2.08±0.43	0.45±0.10
Hexadecane	544-76-3	41.75		57	94	37	0.95±0.18	1.18±0.14	0.88±0.18	0.88±0.09
Dodecanal	112-54-9	35.95	1411	57	87	46	0.88±0.10^*a,b*^	0.74±0.29^*a,b*^	1.42±0.36^*b*^	0.42±0.07^*a*^
Pyrazine, 2,6-diethyl-	13067-27-1	28.68	1235	135	74	51	0.81±0.07	0.74±0.26	1.09±0.18	0.52±0.10
Dodecane	112-40-3	27.33	1202	57	97	50	0.79±0.28	0.52±0.12	0.47±0.20	0.42±0.10
1-Dodecanol	112-53-8	38.38	1477	83	97	35	0.69±0.07	0.17±0.11	3.51±2.44	0.26±0.03
Tetradecane	629-59-4	35.57	1400	57	98	43	0.68±0.05	0.90±0.15	0.87±0.14	0.67±0.13
Heptanal	111-71-7	13.85	904	70	96	45	0.52±0.11	0.65±0.32	1.65±0.58	0.20±0.12
Tridecane	629-50-5	31.57	1301	57	89	57	0.35±0.13	0.76±0.14	0.30±0.18	0.30±0.16
Undecane	1120-21-4	22.77	1101	71	88	47	0.33±0.15	0.49±0.17	0.73±0.17	0.45±0.27
Dodecanoic acid propyl ester	3681-78-5	42.23		102	74	54	0.29±0.04^*a*^	0.01±0.01^*b*^	0.30±0.10^*a*^	0.25±0.04^*a*^
n-Butyl myristate	110-36-1	45.29		102	79	55	0.29±0.04^*a*^	0.03±0.02^*b*^	0.18±0.07^*a,b*^	0.15±0.04^*a,b*^
Oleic acid methyl ester	112-62-9	48.49		55	83	56	0.21±0.15	0.01±0.01	0.57±0.31	0.00±0.00
Methacrolein	78-85-3	6.01		71	95	62	0.20±0.12	0.36±0.23	0.00±0.00	0.00±0.00
Nonadecane	629-92-5	46.21		57	85	52	0.20±0.07	0.26±0.06	0.39±0.29	0.17±0.04
Butanal, 3-methyl-	590-86-3	7.89	703	44	82	60	0.17±0.08	0.06±0.06	0.42±0.31	0.03±0.03
Palmitic acid methyl ester	112-39-0	46.47		74	89	44	0.13±0.10	0.15±0.06	0.76±0.65	0.35±0.02
Methyl myristate	124-10-7	43.93		74	92	48	0.11±0.06	0.04±0.04	0.43±0.39	0.53±0.30
Eicosane	112-95-8	47.25		57	81	58	0.09±0.04^*a,b*^	0.35±0.05^*a*^	0.28±0.12^*b*^	0.04±0.02^*a,b*^
1-Octadecanol	112-92-5	48.06		83	87	53	0.02±0.02	0.47±0.46	0.27±0.09	0.03±0.03
Hexadecanoic acid ethyl ester	628-97-7	47.15		88	79	61	0.00±0.00	0.01±0.01	0.15±0.14	0.00±0.00
Tetradecanoic acid	544-63-8	44.46		73	94	39	0.00±0.00	0.06±0.04	2.61±1.93	0.01±0.01
Total aliphatics							49.27±15.54	56.93±22.57	83.51±23.50	29.50±6.86

**Table 2.** Volatile organic compounds responsible for the differences in the volatile blend emitted by *Dionaea muscipula* before and after insect feedingID numbers reflect the VOC numbers of Table 1 and Fig. 4. SE, jack-knife standard error calculated by cross-validation; VIP, variable importance in the projection.

ID	VOC	VIP ± SE
1	Caryophyllene	1.92548±0.332106
14	*trans*-Verbenone	1.92372±0.257128
6	*p*-Cymene	1.81219±0.274905
7	Limonene	1.80358±0.573093
54	Dodecanoic acid propyl ester	1.73544±0.276104
8	α-Phellandrene	1.708±0.546045
3	Caryophyllene oxide	1.67395±0.320588
19	*cis*-para-2-Menthen-1-ol	1.56589±0.198518
58	Eicosane	1.55199±0.288203
55	n-Butyl myristate	1.50214±0.388094
10	3-Carene	1.48339±0.527291
21	γ-Terpinene	1.38571±0.390942
9	Sabinene	1.36681±0.380447
11	Tricyclene	1.23914±0.1922
16	Camphene	1.18609±0.227142
2	*trans*-Geranylacetone	1.12931±0.523829
28	Phenylethyl alcohol	1.09166±0.751269
37	Hexadecane	1.08186±0.645894
20	*trans*-Pinocarveol	1.07714±0.528759
57	Tridecane	1.06722±0.78692
41	1-Octanol	1.04419±0.909551
13	α-Terpinene	1.00365±0.316166
Total sesquiterpenes		1.90118±0.413713
Total monoterpenes		1.8023±0.410466

## Discussion

A mechanism of insect attraction by *Dionaea muscipula* was already expected by Darwin (1875), but remained investigated experimentally. This work tested the hypothesis that the emission of VOC by the Venus flytrap is involved in the attraction of animal prey. It was demonstrated that *Dionaea* plants emitted a rich bouquet composed of more than 60 VOCs, mainly terpenes, aromatic, and aliphatic compounds including alkanes, carbonyls, alcohols, acids, and esters (Table 1), many of these only if the plants were exposed to light (Fig. 3). The majority of these compounds (with the exception of only two, 1-(1-cyclohexen-1-yl)-ethanone and dodecanoic acid propyl ester) have been described as typical volatile constituents of fruit and flower scents (Knudsen *et al.*, 1993; Rowan *et al.*, 2009; Gonzalez-Mas *et al.*, 2011). The compounds caryophyllene, ocimene, linalool, α-pinene, 6-methyl-5-hepten-2-one, benzyl alcohol, benzaldehyde, and phenylethyl alcohol are particularly widespread, occurring in the floral scent of more than half of the families of all seed plants (Knudsen and Gershenzon, 2006). Nonfloral organs of herbaceous plants, particularly the green leaves, also emit volatiles although usually at lower rates and other composition with a lower portion of terpenes (Loreto and Schnitzler, 2010). The present results strongly suggested that the scent emitted by intact *Dionaea muscipula* was comparable to the scent of fruits and flowers that cause the attraction of insects. In a first work on the VOC emission of carnivorous plants, Jürgens *et al*. (2009) determined a total of 11 VOCs released by isolated *Dionaea muscipula* traps. In consistence with the present study, Jürgens *et al*. (2009) observed the release of benzyl alcohol and traces of 6-methyl-5-hepten-2-one, the monoterpenes linalool, limonene, and ocimene, and the sesquiterpene caryophyllene. However, plant injury, presumably caused by isolating the snap traps, seemed to trigger the emission of compounds typical for abiotic and biotic stress (Borsani *et al.*, 2001; Chen *et al.*, 2003; Munné-Bosch and Peñuelas, 2003; Clarke *et al.*, 2004) such as methyl salicylate, which amounted to 68% of all VOC emitted under this artificial condition (Jürgens *et al.*, 2009). The release of wounding-induced compounds (other than some C6 compounds; Fig. 3) was not observed in the present study. Similarly to this study with the Venus flytrap, a broad spectrum of VOCs consisting of over 50 compounds was emitted by the carnivorous vine *Nepenthes rafflesiana*; the volatile blend consisted of numerous terpenes and aromatic compounds typically found as components of flower scents (Di Giusto *et al.*, 2010).

The present study used *Drosophila melanogaster* as the animal system to investigate its attraction by the blend of volatiles released by the Venus flytrap. *Drosophila melanogaster* is a ubiquitous insect occurring all over the world, from tropical to temperate regions, and is only missing at extremes of altitude and latitude (David and Capy, 1988). Fruit flies mainly feed on plant material, particularly ripe and rotten fruits rich in sugars such as sucrose, glucose, and fructose (Burke and Waddell, 2011). The olfactory system of *Drosophila melanogaster* is very well studied at the molecular and cellular level and has been used for several plant—insect interaction studies, including the attraction by carnivorous plants (Di Giusto *et al.*, 2010). The current work’s olfactory choice bioassays demonstrated that the VOCs released by the Venus flytrap attracted starved flies very strongly (Fig. 2C, D). Considering the high portion of animals making a decision towards the plant (94% males, 76% females), attraction by *Dionaea* was even slightly stronger than by their familiar food odour (67% of males and 68% of females were attracted; Fig. 2B). Flies were most probably expecting a food source, as some of the odours released by *Dionaea* were components of ripe and rotten fruits, the natural food preference of *Drosophila*. Nonstarved fruit flies did not show any movement in the Y-tube (data not shown). This finding is in accordance with studies on the attraction of hungry *Drosophila* (Larsson *et al.*, 2004; Semmelhack and Wang, 2009; Becher *et al.*, 2010; Lebreton *et al.*, 2012; Steck *et al.*, 2012) by volatiles, including acetic acid, 2-phenylethanol, benzaldehyde, and hexenal which were also components of the volatile *Dionaea* scent.

Previous work on fruit flies clearly indicated that several of the volatiles contained in the carnivorous plant’s VOC blend are potential ligands of olfactory receptorsm including products of the LOX reaction (hexenal, hexanol), aromatic (acetophenone, phenylethanol, benzaldehyde), and aliphatic compounds (acetic acid, octanol, methyl acetate) as well as terpenes (e.g. caryophyllene) (Stensmyr *et al.*, 2003; Zhu *et al.*, 2003; Becher *et al.*, 2010; Tunstall and Warr, 2012). Also other insect species are known to react on some of the oxygenated compounds emitted by *Dionaea* such as LOX products, linalool, benzaldehyde, benzyl alcohol, 2-phenylethanol, and methyl benzoate (Bruce *et al.*, 2005; Jhumur *et al.*, 2008). It is still a matter of debate if single compounds are responsible for specific plant location by insects or if a distinct ratio between constituent volatiles is decisive (Bruce *et al.*, 2005). Insects must detect their host plants even against a background of volatiles released by other plant species. Although there are some exceptions where host recognition occurs via single compounds (Blight *et al.*, 1995), most phytophagous insects seem to detect a specific blend of ubiquitous volatiles emitted by the host plant. This specific blend is detected against the background noise of other blends most probably because of the specificity of the plant volatile olfactory receptor neurons (Bruce *et al.*, 2005). It is assumed that *Dionaea* attracts its prey via emission of such ubiquitous VOCs as this would enable the carnivorous plant to cover a broad range of insects. Fruit-feeding insects such as *Drosophila* might preferentially be attracted by oxygenated compounds often present in ripe or rotten fruit, whereas pollinators would additionally be attracted by terpenoids, also released by the Venus flytrap (Knudsen and Gershenzon, 2006; Dudareva *et al.*, 2013).

It is well known that VOC emission by plants can be influenced by N nutrition. Several studies have shown that VOC emissions positively correlate with N availability in the soil (Harley *et al.*, 1994; Lerdau *et al.*, 1995). To test whether the plant N status affected the attractiveness of the Venus flytrap for *Drosophila melanogaster*, this work fed *Dionaea muscipula* with insect powder which improved its N status, as seen from the considerably higher total N contents, increased rates of photosynthesis, and 2–3-times higher relative growth rates than in unfed plants (Kruse *et al.*, 2013). As a consequence, the profile of VOCs emitted by the plants was significantly altered (Fig. 4, Table 2, and Supplementary Fig. S1). The observed reduced emission of terpenoids, however, did not affect the Venus flytrap’s attractiveness on fruit flies. Given that the profile of benzenoids and aliphatic compounds was not changed in fed *Dionaea muscipula*, this work further concludes that particularly these compounds, which are also constituents of ripe and rotten fruits, are recognized by *Drosophila melanogaster*. This assumption is supported by findings indicating that the olfactory receptors of *Drosophila melanogaster* (Zhu *et al.*, 2003; Becher *et al.*, 2010; Tunstall and Warr, 2012), but also of other insects (Bruce *et al.*, 2005; Gols *et al.*, 2012), are stimulated by such oxygenated compounds.

Because several VOCs were emitted from *Dionaea muscipula* only during the light period (Fig. 3), this work tested whether dark-exposed Venus flytraps also lured fruit flies. Indeed, in accordance with the hypothesis that VOCs act as olfactory signal for insects, dark exposed *Dionaea muscipula* did not strongly attract *Drosophila melanogaster*; only male animals were weakly attracted by the plants (Fig. 2f). It is proposed that reduced release of VOCs during night is the result of (i) the lack of plant internal VOC pools which would cause VOC emission also during darkness, (ii) reduced stomatal conductance during night inhibiting the emission particularly of oxygenated compounds (Loreto and Schnitzler, 2010), and (iii) reduced leaf temperature (from 34 °C during day to 21 °C during night) which slows biosynthesis and emission of terpenes (Guenther *et al.*, 1993; Kreuzwieser *et al.*, 2002) and oxygenated compounds (Cojocariu *et al.*, 2004). The reduced terpenoid emission during night (Fig. 3) can furthermore be explained by the strong light-dependence of terpenoid biosynthesis localized in the chloroplasts (Rodríguez-Concepción, 2006; Tholl and Lee, 2011; Loreto and Schnitzler, 2010). Biosynthesis of sesquiterpenes is localized in the cytosol (Tholl and Lee, 2011) but in green plant tissues also depends on the light-regulated supply of photoassimilates to the mevalonate pathway.

The present results on olfactory signals of the Venus flytrap together with published literature on attraction via nectary cues (Bennett and Ellison, 2009) and visual cues (Ichiisi *et al.*, 1999) give a consistent answer to Charles Darwin’s question on the existence of an animal attraction mechanism of *Dionaea muscipula.* A complex mixture of ubiquitous volatile compounds emitted by the Venus flytrap might serve as a first signal attracting prey insects from distant locations and leading them closer to the plant. At a closer distance, a combination of olfactory and visual signals and nectaries channels the insect into the deadly traps.

## Supplementary material

Supplementary data are available at *JXB* online.

Supplementary Fig. S1. Two-dimensional score and scaled-loading plots of partial least square regression computed with VOC emission rates emitted from unfed and fed *Dionaea muscipula* plants

Supplementary Data
