# The Venus flytrap attracts insects by the release of volatile organic compounds

**DOI:** 10.1093/jxb/erv242

**Published:** 2015-05-21

**Authors:** Jürgen Kreuzwieser, Ursel Scheerer, Jörg Kruse, Tim Burzlaff, Anne Honsel, Saleh Alfarraj, Plamen Georgiev, Jörg-Peter Schnitzler, Andrea Ghirardo, Ines Kreuzer, Rainer Hedrich, Heinz Rennenberg

**Affiliations:** ^1^ Professur für Baumphysiologie, Institut für Forstwissenschaften, Albert-Ludwigs-Universität Freiburg, Georges-Köhler-Allee Geb. 053/054, 79110 Freiburg, Germany; ^2^ Professur für Forstzoologie und Entomologie, Institut für Forstwissenschaften, Albert-Ludwigs-Universität Freiburg, Tennenbacher Strasse 4, 79085 Freiburg, Germany; ^3^ Zoology Department, College of Science, King Saud University, Riyadh 11451, Saudi Arabia; ^4^ Fly Facility, Max Planck Institute of Immunobiology and Epigenetics, Stübeweg 51, 79108 Freiburg, Germany; ^5^ Research Unit Environmental Simulation (EUS), Institute of Biochemical Plant Pathology, Helmholtz Zentrum München, Ingolstädter Landstr. 1, 85764 Neuherberg, Germany; ^6^ Lehrstuhl für Botanik I, Julius-von-Sachs-Institut für Biowissenschaften, Julius-von-Sachs-Platz 2, 97082 Würzburg, Germany

*Journal of Experimental Botany*, Vol. 65, No. 2, pp. 755–766, 2014

doi:10.1093/jxb/ert455

The Acknowledgements section reads that this study was financially supported by the Distinguished Scientists Program, King Saud University, Riyadh, Saudi Arabia (grants to HR and RH).

In fact, this work on Rainer Hedrich’s side was funded by an ERC grant.

The corrected Acknowledgements section is reproduced below.

**Acknowledgements**

This study was financially supported by the Distinguished Scientists Program, King Saud University, Riyadh, Saudi Arabia (grants to HR) as well as funding from the European Research Council under the European Union’s Seventh Framework Programme (FP/20010–2015)/ERC Grant Agreement number 250194-Carnivorom (grant to RH).

