# Supplementary material for: The Venus flytrap attracts insects by the release of volatile organic compounds
Source: J Exp Bot. 2014 Jan 13;65(2):755–66. doi: 10.1093/jxb/ert455 (PMC3904726; doi:10.1093/jxb/ert455)
Supplement: Supplementary Data [file supp_65_2_755__index.html]

The Venus flytrap attracts insects by the release of volatile organic compounds — The Venus flytrap attracts insects by the release of volatile organic compounds — Supplementary Data 

# The Venus flytrap attracts insects by the release of volatile organic compounds

## Supplementary Data

Data files

**Files in this Data Supplement:**

- Supplementary Data - Supplementary Data
