# Supplementary material for: The Venus flytrap attracts insects by the release of volatile organic compounds
Source: J Exp Bot. 2014 Jan 13;65(2):755–66. doi: 10.1093/jxb/ert455 (PMC3904726; doi:10.1093/jxb/ert455)
Supplement: Supplementary Data [file supp_ert455_jexbot106740_file001.pdf]

The Venus flytrap attracts insects by the release of volatile organic compounds

Jürgen Kreuzwieser, Ursula Scheerer, Jörg Kruse, Tim Burzlaff, Anne Honsel, Saleh Alfarraj, Plamen Georgiev, Jörg-Peter Schnitzler, Andrea Ghirardo, Ines Kreuzer, Rainer Hedrich, and Heinz Rennenberg

Supplemental Figure S1. Two-dimensional (A) score and (B) scaled-loading plots of partial least square regression (PLSR) computed with VOC emission rates emitted from unfed and fed *D. muscipula* plants. Plants were divided into treatment group (blue) and control group (red). Circles indicate VOC emission rates emitted from treated (O) and control (O) plants at day 0 (unfed), triangles their respective emission rates 4 days after, when treated plants (▲) were fed with insect powder and control plants (▲) remained unfed. The explained variance (in percentage) and the number of principal component are reported in x- and y-axes in both (A) and (B) plots. Ellipse in (A) indicates the tolerance based on Hotelling's T^2 with significance level of 0.05. Numbers and letters in (B) reflect the compounds listed in Table 1, "Feeding" (in blue) show the Y-loading weight. The outer and inner ellipses in (B) indicate 100% and 75% explained variance, respectively.

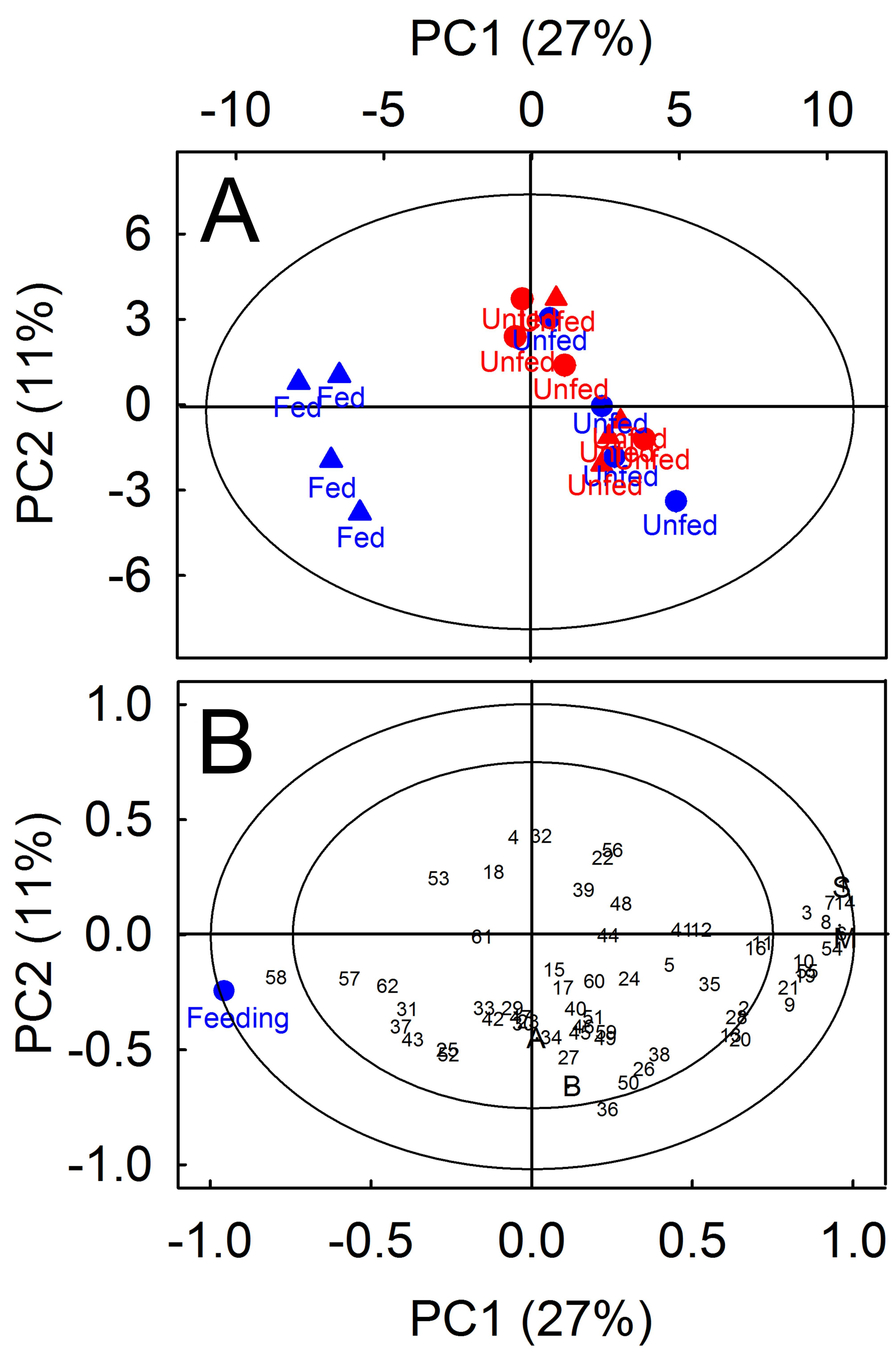

PC1 (27%)
-10
-5
0
5
10
A
6
3
0
-3
-6
PC2 (11%)
Fed
Fed
Fed
Fed
Unfed
Unfed
Unfed
Unfed
Unfed
Unfed
B
1.0
0.5
0.0
-0.5
-1.0
Feeding
-1.0
-0.5
0.0
0.5
1.0
PC1 (27%)
